# The Interplay between Liver Sinusoidal Endothelial Cells, Platelets, and Neutrophil Extracellular Traps in the Development and Progression of Metabolic Dysfunction-Associated Steatotic Liver Disease

**DOI:** 10.3390/jcm13051406

**Published:** 2024-02-29

**Authors:** Iulia Minciuna, Madalina Gabriela Taru, Bogdan Procopet, Horia Stefanescu

**Affiliations:** 1 Regional Institute of Gastroenterology and Hepatology “Prof. Dr. Octavian Fodor”, 400394 Cluj-Napoca, Romaniahoria.stefanescu@irgh.ro (H.S.); 2 Deaprtment IV, “Iuliu Hatieganu” University of Medicine and Pharmacy, 400347 Cluj-Napoca, Romania

**Keywords:** endothelial dysfunction, neutrophil extracellular traps, platelets, MASLD

## Abstract

Metabolic dysfunction-associated steatotic liver disease (MASLD) represents a societal burden due to the lack of effective treatment and incomplete pathophysiology understanding. This review explores the intricate connections among liver sinusoidal endothelial cells (LSECs), platelets, neutrophil extracellular traps (NETs), and coagulation disruptions in MASLD pathogenesis. In MASLD’s early stages, LSECs undergo capillarization and dysfunction due to excessive dietary macronutrients and gut-derived products. Capillarization leads to ischemic changes in hepatocytes, triggering pro-inflammatory responses in Kupffer cells (KCs) and activating hepatic stellate cells (HSCs). Capillarized LSECs show a pro-inflammatory phenotype through adhesion molecule overexpression, autophagy loss, and increased cytokines production. Platelet interaction favors leucocyte recruitment, NETs formation, and liver inflammatory foci. Liver fibrosis is facilitated by reduced nitric oxide, HSC activation, profibrogenic mediators, and increased angiogenesis. Moreover, platelet attachment, activation, α-granule cargo release, and NETs formation contribute to MASLD progression. Platelets foster fibrosis and microthrombosis, leading to parenchymal extinction and fibrotic healing. Additionally, platelets promote tumor growth, epithelial–mesenchymal transition, and tumor cell metastasis. MASLD’s prothrombotic features are exacerbated by insulin resistance, diabetes, and obesity, manifesting as increased von Willebrand factor, platelet hyperaggregability, hypo-fibrinolysis, and a prothrombotic fibrin clot structure. Improving LSEC health and using antiplatelet treatment appear promising for preventing MASLD development and progression.

## 1. Introduction

Reaching an estimated global prevalence of 25% [1], metabolic dysfunction-associated steatotic liver disease (MASLD) is the leading cause of chronic liver disease and an emerging risk factor for cirrhosis and hepatocellular carcinoma (HCC) development in the Western world [2]. MASLD consists of a broad spectrum of stages, ranging from simple steatosis to steatohepatitis (MASH) and cirrhosis, and along with the associated metabolic comorbidities, renders to become a significant burden to society in terms of low quality of life and an expanding need for healthcare resources. This burden is further increased by the absence of an efficient treatment and the lack of a full understanding of the pathophysiological mechanism of disease progression.

So far, the multiple hit hypothesis of MASH pathogenesis identifies multiple insults such as insulin resistance, nutritional factors, and endotoxemia through gut microbiota that act together on genetically predisposed subjects to induce MASLD [3]. Hepatic inflammation and subsequent fibrosis, through inflammatory response in Kupffer cells (KC) and fibrotic response in hepatic stellate cells (HSC), are the most studied pathways of disease progression. Liver sinusoidal endothelial cells (LSECs) seem to be a key player in promoting inflammation and fibrosis. However, the triggering factors for inflammation still need to be fully understood. More recently, a novel insight into the mechanism of inflammation initiation underlines the key role of neutrophils extracellular traps (NETs) in MASH progression and HCC development [4]. NETs are neutrophil components implicated in numerous diseases like venous or arterial thrombosis, sepsis, or systemic inflammatory conditions. NETs, triggered by free fatty acids (FFA), interact with monocytes, releasing inflammatory cytokines [4]. Nevertheless, this mechanism cannot be entirely explained by the presence of FFA, and an alternative pathway of NETs expression can be hypothesized. 

Indeed, by KC activation and conversion of HSCs into myofibroblasts, platelets have recently been shown to be involved in MASLD pathogenesis and progression in animal models [5]. New insights show that platelets are the first extrahepatic cells that arrive in the liver as early as 4 weeks after the beginning of a high-fat diet, which coincides with the nonalcoholic fatty liver-nonalcoholic steatohepatitis transition in animal models [5]. Moreover, in septic patients, activated platelets by bacterial lipopolysaccharide (LPS) attach to neutrophils to activate them, to release NETs and subsequently to capture bacteria [6]. As intestinal dysbiosis with secondary endotoxemia and bacterial translocation was shown to be one of the pathogenetic mechanisms of MASH, platelets may accentuate the inflammatory milieu by favoring the development of NETs [7]. 

Nevertheless, the role of platelets through NETs formation is not clearly understood. Indeed, some patients with MASH present different histological vascular alterations also encountered in other conditions, such as vascular liver diseases, for which the main mechanism involved is thrombosis. These findings suggest a possible common pathophysiological pathway through platelet activation of coagulation in both diseases. 

This review aims to characterize the interplay between LSEC, platelets, NETs, and the coagulation disturbances in the pathogenesis of MASH.

## 2. Liver Sinusoidal Endothelial Cells in MASLD

### 2.1. Liver Sinusoidal Endothelial Cells’ Roles

In addition to serving as a physical barrier regulating molecular exchanges between the liver parenchyma and circulation, the liver endothelium, primarily represented by the highly specialized liver sinusoidal endothelial cells (LSECs), is also involved in several pathophysiological processes, such as inflammation, angiogenesis, the regulation of vascular tone and platelet function, the hepatic immune response, and metabolic homeostasis [8,9].

LSECs are situated at the interface between blood derived from the gut and visceral adipose tissue on one side and other liver cells on the other. On the sinusoidal side, LSECs are exposed to blood-circulating nutrients, hormones, bile acids, and oxygen. In contrast, on the abluminal side, LSECs directly interact with HSCs and hepatocytes, which are involved in glucose and lipid metabolism [10]. LSECs have a unique phenotype since they lack a basement membrane and exhibit open fenestrae without a diaphragm, rendering them the most permeable endothelial cells and endowing them with the highest endocytosis capacity of all human cells [11]. In physiological conditions, LSECs contribute to regulating the vascular tone and are involved in the secretion of molecules with vasoactive properties, such as nitric oxide (NO) [12]. Moreover, by maintaining HSC quiescence, LSECs can prevent intrahepatic vasoconstriction and the development of liver fibrosis [7].

The LSECs fenestrae connects the space of Disse to the sinusoidal side of hepatocytes, allowing lipoproteins, chylomicron fragments, and other macromolecules from circulating blood to enter the space of Disse and be taken up by hepatocytes. The size and number of fenestrae vary within different hepatic lobule zones, changing upon the physiological or pathological states, including fasting, oxygen availability, or pathological states [13,14]. Additionally, LSECs act as antigen-presenting cells (APCs), influencing immune homeostasis by releasing cytokines and activating immune cell pathways [15]. LSEC also regulates intrahepatic coagulation through mechanisms such as the direct generation of both pro- and anticoagulant factors, neutrophils recruitment and activation, and the interaction with platelets [16,17,18].

### 2.2. Liver Sinusoidal Endothelial Cells’ Capillarization

Liver injuries, both acute and chronic, induce functional and phenotypic changes in all liver cells. LSECs acquire a capillarized phenotype consisting of the loss of fenestrae and a basement membrane development, which impedes the proper oxygenation of hepatocytes and the common exchanges with the extracellular space. The resulting apoptotic and necrotic hepatocytes release damage-associated-molecular-patterns (DAMPs) that activate HSC and enhance their pro-proliferative, pro-contractile, and procollagen-synthetic features [19,20]. As part of the capillarization process, and influenced by paracrine signals from other sinusoidal cells, KCs polarize towards a pro-inflammatory phenotype, thereby activating the immune response and inflammation processes [21]. Increased extracellular matrix deposition activates HSCs, raising intrahepatic vascular resistance [22].

LSEC endothelial dysfunction arises from an abnormal imbalance between relaxing and contracting factors, hindering blood vessel caliber adjustment to increased blood flow [23]. Both capillarization and endothelial dysfunction occur early in the MASLD natural history [10]. Excessive dietary macronutrients such as lipids, carbohydrates, and gut-derived products can trigger sinusoidal capillarization. Cogger et al. found LSEC porosity inversely correlated with dietary fat intake, and fenestrae diameter inversely associated with protein or carbohydrate intake in mice exposed to varying macronutrient diets [24]. Furthermore, a number of LSEC exhibited a negative correlation with the abundance of Bacteroidetes in the gastrointestinal tract while showing a direct correlation with the abundance of Firmicutes, both of which outline microbiota’s role in the MASLD pathogenesis [24]. Moreover, gut-derived products can also impact LSEC’s phenotype since a single injection of endotoxin in rats reduced the number and diameter of their fenestrae [25].

Three mechanisms may explain how capillarized LSEC can favor steatosis development in MASLD patients. The first one refers to the preventing effect of capillarized LSEC in releasing very-low-density lipoprotein (VLDL) from hepatocytes, resulting in the accumulation of lipids within the liver [7]. However, because chylomicron remnants are necessary for hepatocytes to produce VLDL, capillarized LSEC’s inhibitory effect on chylomicron remnant entry into hepatocytes stimulates de novo lipogenesis, which eventually results in hepatic steatosis as a compensatory mechanism [7,26]. Liver steatosis also contributes to endothelial dysfunction through different mechanisms as well. Due to the compression of the sinusoidal lumen by steatotic hepatocytes, there is an increase in intrahepatic vascular resistance, impeding the vascular bed from properly expanding in response to increased blood flow [27,28]. Moreover, in vitro studies have shown that co-culturing LSECs with ox-LDL leads to the downregulation of eNOS expression through the ox-LDL receptor, while the exposure of primary LSECs to palmitic acid can reduce NO bioavailability through LSEC overexpression of a NO consuming enzyme [29,30]. Moreover, this excessive exposure to ox-LDL can trigger a decrease in fenestrae diameter and in LSEC porosity [29]. A third mechanism refers to the impairment of insulin-dependent vasodilation through the downregulation of eNOS activity and the overexpression of inducible nitric oxide synthase (iNOS), resulting in increased nitro-oxidative stress and potentially leading to endothelial dysfunction [31,32]. 

### 2.3. Endothelial Dysfunction and Angiogenesis during MASLD

Angiogenesis, the formation of new vessels from pre-existing ones, plays a crucial role in MASLD progression. Several mechanisms contribute to angiogenesis during MASH, including tissue hypoxia, HSC activation, endothelial dysfunction, inflammation, and lipotoxicity.

Steatosis induces angiogenesis by creating a hypoxic microenvironment triggered by elevated oxygen consumption due to increased fatty acid metabolism and decreased bioavailability, as steatotic hepatocytes exert mechanical pressure on sinusoids [28,33]. However, hepatic neovascularization primarily occurs in MASH patients, not in those with simple steatosis or normal liver [33,34]. Moreover, in MASLD patients, hypoxia may be more pronounced than in other chronic liver diseases because the primary injury occurs in the perivenular zone, which is highly susceptible to hypoxia compared to periportal areas [35]. Furthermore, within the liver, angiogenesis and fibrosis show a close spatial association. The distinctive collagen deposition in MASH is characterized by peri-sinusoidal fibrosis, primarily in zone 3, and appears to coincide with the site of liver angiogenesis observed in both human subjects and animal models of MASH [36,37]. This alignment could be attributed to the response to hypoxia of HSCs within developing fibrotic septa, leading to HSC expression of pro-angiogenic factors like s VEGF and ANG-1 [38]. 

Endothelial dysfunction characterized by the loss of LSEC NO-inhibitory effect on HSC is another factor that contributes to HSC activation and, thus, to angiogenesis [12]. Dysfunctional LSECs contribute to pathologic angiogenesis by releasing pro-angiogenic factors such as VEGF, TGF-β, angiopoietin-2, and adipokines [7]. Moreover, chronic injury alters chemokine signaling in LSECs, promoting a CXCR7-driven profibrogenic and pro-angiogenic program [38].

Inflammation is a key inducer of angiogenesis in MASH, maintaining tissue hypoxia and activating hypoxia-inducible factor 1α (HIF1α)-dependent genes. Pro-inflammatory mediators and cytokines stimulate angiogenesis by inducing HIF-1α transcriptional activity, VEGF production, and activating the MAPK/ERK pathway involved in cell migration and angiogenesis [39]. Moreover, during MASH, infiltrating monocytes differentiate into monocyte-derived macrophages that are distributed in fibrotic areas, where they stimulate angiogenesis through VEGF and MMP9 production [40,41]. This was confirmed by Ramachandran et al.’s study, in which they showed, using a single-cell sequencing approach, that scar-tissue-associated macrophages exhibit increased expression of pathways related to fibrogenesis and angiogenesis [42]. Furthermore, Ehling et al. showed that preventing monocyte infiltration into the liver halted fibrosis-related angiogenesis but not the development of fibrosis [40].

Inflammation can also stimulate angiogenesis indirectly through the action of angiopoietin-2, a key regulator of angiogenesis that, under physiological conditions, maintains vascular stability and quiescence, while during inflammation, it promotes angiogenesis [43]. In the study of Lefere et al., serum angiopoietin-2 levels were elevated in MASH patients and correlated with liver steatosis, inflammation, and hepatocyte ballooning but not with liver fibrosis [36]. The interaction between inflammation and angiogenesis is bidirectional since the inhibition of angiogenesis using anti-VEGFR2 antibody improved liver vasculature and reduced liver inflammatory gene expression [44]. Lipotoxicity is another link to angiogenesis during MASH, as in vitro studies have demonstrated that hepatocytes exposed to excessive amounts of saturated FFA produce microvesicles with a pro-angiogenic activity [45]. 

### 2.4. Endothelial Dysfunction and Liver Inflammation

Increasing evidence outlines the role of LSEC endothelial dysfunction in promoting inflammation in MASLD patients. Endothelial dysfunction and LSEC capillarization are early events in the natural history of MASLD, occurring before liver inflammation and promoting its development [46]. Mechanisms involve decreased phosphorylation of Akt-dependent eNOS and reduced NO bioavailability, crucial for maintaining KCs quiescence [47]. KCs are resident macrophages located within the liver sinusoids that are in close contact with LSEC, and that can be activated by various factors, including pathogen-associated molecular patterns such as lipopolysaccharide (LPS), DAMPs secreted by damaged hepatocytes, and lipids including FFAs, ceramides, and oxidized lipoproteins [48,49,50]. Activation of KCs promotes inflammation by triggering the induction of nuclear factor-κB and upregulation of pro-inflammatory factors TNF-α and IL-6, as shown in murine experiments [51]. This process is enhanced as activated KCs promote LSEC capillarization, leading to leukocyte recruitment, adhesion, and transmigration.

As MASLD progresses, LSECs acquire a pro-inflammatory phenotype, overexpressing adhesion molecules such as ICAM-1, VCAM-1, E-selectin, and VAP-1 at the surface of LSECs and producing inflammatory mediators, including TNFα, IL-6 and IL-1 [52]. Among the different overexpressed adhesion molecules, VCAM-1 stimulates the development of a firm interaction between leukocytes and the endothelium, leading to the formation of inflammatory hubs [53]. In MASLD, leukocytes migrate from the circulation into the liver parenchyma, forming inflammatory foci that drive disease progression. Activated LSEC recruit leukocytes via multi-step adhesion cascades involving different cytokines and receptors on the surface of leukocytes and LSEC.

Compared to other solid organs, sinusoidal endothelial cells within the liver have a distinct phenotype marked by reduced expression of p-selectin and lower levels of von Willebrand factor (vWF), rendering the liver a specialized environment for platelet-endothelial interactions [54]. This is also reflected by the different signaling combinations that govern the recruitment of immune cells across the sinusoidal bed, as within the liver, platelets compensate for the lack of expression of adhesion molecules such as selectins by assisting in leukocyte recruitment during inflammation [55].

The interaction with LSEC grants platelets access to the liver parenchyma and represents a priming process for initiating liver repair upon injury and liver regeneration. However, this interaction also contributes to the development of liver inflammation and hepatic damage. The recruitment of platelets at the site of liver damage is a dynamic process that seeks to localize and delimitate the injured area by facilitating the adhesion of platelets to the altered LSECs [56]. However, during endotoxemia, platelets–LSEC interactions precede and drive liver leucocyte recruitment and result in hepatic damage. This is a self-perpetuating process of microvascular dysfunction and hepatocellular damage as leukocytes per se can lead to platelet recruitment within the liver [57]. Once recruited, platelets interact with neutrophils, promoting neutrophil extracellular trap development, intensifying liver inflammation, microthrombosis, and portal hypertension [58].

Another factor that contributes to the interplay between endothelial dysfunction and liver inflammation in MASH patients is the defect LSEC autophagy. This abnormal cell-specific autophagy significantly decreases the number and porosity of LSEC fenestrae in mice after mild acute liver injury and increases the expression of pro-inflammatory chemokines such as C-C motif chemokine ligand (CCL) 2, CCL5, and vascular cell adhesion molecule (VCAM-1) which further promotes inflammation in murine models of MASLD [59].

### 2.5. Endothelial Dysfunction and Liver Fibrosis

Endothelial dysfunction is an early event in the course of MASLD, preceding fibrosis in MASH animal models, and contributing significantly to its development. Following capillarization, LSEC can lead to the development of liver fibrosis by losing their ability to maintain HSC quiescence via NO production [12]. In a murine study with rats on a high-fat diet, simvastatin increased eNOS expression, ameliorating liver fibrosis, while the inhibition of eNOS using L-NAME blunted the protective effect of LSEC upon HCS quiescence [60,61]. LSECs can promote liver fibrosis by releasing profibrogenic molecules like TGF-β, Hedgehog molecules, laminin, and fibronectin, activating HSCs to produce the extracellular matrix [7]. Activated HSCs release Hedgehog molecules, thereby reinforcing their own activation and promoting LSEC capillarization [62].

In MASH patients, liver angiogenesis supports fibrosis through mechanisms like tissue hypoxia, hepatocyte-derived microvesicles, angiopoietin-2 expression, and elevated leptin levels with a direct pro-fibrotic effect by upregulating TGF-β in LSECs and KCs [7]. In the study of Kitade et al., a rat model of MASH showed no angiogenesis or fibrosis development in the absence of leptin signaling [37]. The interconnection between the two is also indirectly outlined in different studies showing that inhibiting liver angiogenesis can prevent MASH-related fibrosis. By inhibiting either pro-angiogenic macrovesicles release from steatotic hepatocytes or their binding to the target cells, MASH murine models were protected from angiogenesis development and had decreased liver fibrosis [45]. 

## 3. The Roles of Platelets in MASLD

Platelets play a crucial role in orchestrating and amplifying inflammatory responses to various injuries in the liver [55]. Moreover, preliminary studies have shown that in MASH, antiplatelet treatment can lead to less severe histological features of steatohepatitis and a slower progression to advanced fibrosis [63], thus suggesting a potential role of platelets in MASLD pathogenesis. The interaction between platelets and liver homeostasis is complex and bidirectional. While the liver facilitates platelet production, platelets play a crucial role in liver hemostasis, maintaining vascular integrity, regulating immunity, and supporting the function of both parenchymal and non-parenchymal liver cells through the release of the biologically active substances included in their granules, such as growth factors, i.e., VEGF, hepatocyte growth factor (HGF) and platelet-derived growth factor (PDGF), and inflammatory molecules like CXCL1, CXCL4, CXCL5, and CXCL7 [64]. 

In response to damage, platelets adhere to the immobilized vWF and subendothelial collagen via their glycoprotein (GP) receptors GPIb-V-IX and GPVI [65]. Subsequently, activated platelets change their GPIIb/IIIa receptor conformation, which allows them to bind to fibrinogen and initiate other platelet-platelets interaction and thrombus formation [65]. The main components within serum originating from platelet-initiated processes such as coagulation, growth, and wound healing are derived from intracellular sources. Circulating, resting platelets contain multiple storage granules, namely alpha-granules (α-granules), dense granules (δ-granules), lysosomes, and microparticles. After adhesion, they release granule contents, including proteins, growth factors, cytokines, and bioactive lipids, promoting immune cell recruitment, thrombogenesis, and sealing tissue gaps [66]. Moreover, activated platelets interact with leukocytes through P-selectin or granule content release, activating and recruiting leukocytes [67]. Conversely, leukocytes activate platelets and the coagulation cascade, leading to “immunothrombosis”. Despite limiting pathogen spread and enhancing its clearance, immunothrombosis may contribute to endothelial dysfunction and organ damage [64]. During the process of injury resolution, platelets play an important role in stimulating regeneration and restoring homeostasis by releasing chemokines, angiogenic factors, and growth factors [68].

The Malehmir et al. group showed in a murine study that platelets are the first non-resident cells that infiltrate the liver in early MASH, an event that marks the transition from simple steatosis to steatohepatitis. In terms of platelet receptors involved in MASH pathogenesis, they have shown that the GPIIb subunit of the platelet fibrinogen receptor GPIIb/IIIa, as well as platelet integrin α2β3 binding motif of fibrinogen, are not involved in the natural history of the disease [5]. Thus, platelet aggregation does not contribute to MASH, but instead, the platelet attachment and activation via platelet-derived GPIbα receptors are the major players in MASH development, as confirmed by the improvement in steatosis, hepatic injury, fibrosis, and leukocyte infiltration when this receptor is blocked [5]. Moreover, this anti-GPIbα antibody treatment further decreases the degree of cytokine and chemokine production by KCs [5].

Another important platelet receptor involved in both early and late MASH pathogenesis that mediates the interaction between platelets and KCs is the CD44 receptor. The attachment of platelets to the extracellular matrix, in particular to hyaluronan via their CD44 receptor, is essential for establishing direct contact with KCs and triggering the inflammatory response associated with metabolic stress [69]. This is supported by studies using genetic and pharmacological inhibition of CD44 and hyaluronidase showing a decrease in hepatic accumulation of KCs and platelets, which finally leads to reduced inflammation and improved NAFLD activity score [69].

Upon being activated, platelets discharge the content of their granules, which encompass growth factors, inflammatory mediators, and coagulation factors. α-granules, the most abundant type, store adhesive molecules (i.e., vWF and fibrinogen), growth factors (i.e., PDGF, fibroblast growth factor, epidermal growth factor (EGF), HGF, insulin-like growth factor 1, and TGFβ), and inflammatory and angiogenic mediators ((i.e., VEGF-A and -C), angiopoietin-1, and sphingosine-1-phosphate (S1P)), influencing immune cells, endothelial cells, stromal cells, and tissue responses [70]. Moreover, the growth factors can favor within the liver cell-type- and function-specific proliferation and immunomodulation [66]. Platelet δ-granules, although less abundant, constitute a cluster of lysosome-related organelles that accumulate and sequester nucleotides via multidrug-resistance protein 4 and also store transmitters (i.e., serotonin, epinephrine, histamine) involved in liver regeneration, vascular function, and cancer progression [71,72,73,74]. The third category of granules, platelet-lysosomes, are rich in carboxypeptidases and other proteases that control tissue responses and inflammatory processes [69].

Although platelet granule-derived molecules are increased in MASLD, the group of Malehmir et al. concluded that it is actually platelet α-granules that play an active role in the pathogenesis of MASH [5]. They showed that mice with the genetic absence of α-granules that underwent a MASH diet, although having a similar weight gain and intrahepatic platelets numbers, they eventually had better glucose tolerance, lower NAS, decreased cholesterol and aminotransferases levels [5]. Therefore, they showed that apart from their adhesion to KCs, platelets degranulation is also an important step in the development and natural history of MASH. The role of platelets in MASH pathogenesis is also outlined by the effect of the pharmacological inhibitors of platelets activation (i.e., aspirin/clopidogrel) that improve liver steatosis, reduce inflammatory infiltrate, NAFLD activity score, and may decrease the incidence of MASH-induced HCC development [69].

### 3.1. Platelets and Liver Fibrosis

As a result of activation and adhesion, platelets release a variety of cellular mediators from their granule cargo, including TGF, PDGF-B, CXCL4, vWF, and platelet-derived S1P which stimulates HSC to increase collagen secretion, proliferation, migration, and transformation into myofibroblasts [75]. Animal studies have demonstrated that S1P activates and proliferates HSCs, while the accumulation of platelets and platelet-derived chemokine CXCL4 in fibrotic areas in chronic liver disease patients supports in vitro findings that platelet-derived CXCL4 induces HSC proliferation and chemotaxis [65,69]. Another granule cargo content, PDGF-B, activates HSCs, promoting liver fibrosis in mouse models of biliary fibrosis, with antiplatelet treatment demonstrating the ability to decrease fibrosis progression [76]. 

Microthrombi within the liver’s vasculature were seen in fibrotic regions in both human explants and animal models [77,78]. This observation led to the conclusion that coagulation activation can promote fibrosis development and progression through local ischemia and cell death, leading to “parenchymal extinction” as well as through the effect of coagulation proteases such as thrombin and factor Xa upon HCS activation [79,80].

However, other studies suggested that the platelets may contribute to limiting and suppressing HSC activation. The inhibitory effect of platelets on HSC activation is mediated through the cAMP pathway, which is initiated by direct contact with ATP-enriched granules of adhesive platelets. Additionally, the effect is mediated by HGF released from platelets granules [81]. HGF inhibits the activation of ERK1/2 and JNK1, reduces the production of TGF-β and type I collagen, and induces apoptosis in activated HSCs and portal myofibroblasts [82,83]. In addition to increased collagen production, HGF stimulates fibrinolysis by upregulating matrix-metalloproteinases [84].

### 3.2. Platelets and Hepatocellular Carcinoma

MASLD-to-HCC transition is a complex process composed of several mechanisms involved in immune and inflammatory responses, oxidative stress, DNA damage, and autophagy [85]. Among the different factors that can contribute to MASH-to-HCC transition the environmental modifiers, namely diet and lifestyle, are some of the leading actors as they can induce metabolic stress in hepatocytes, resulting in increased levels of ROS, oxidative and endoplasmic reticulum stress, together with the development of metabolic reprogramming [86,87]. These events contribute to increased cell death, DNA damage, compensatory hepatocyte proliferation, enhanced immune cell activation with subsequent HSCs activation and liver fibrosis, all of which contribute to a pro-carcinogenic environment [85,88]. In instances where anti-tumor immune surveillance fails, pre-malignant lesions may develop, culminating in HCC [89].

Platelets play a multifaceted role in HCC development and progression. Two clinical studies performed in HCC patients revealed a positive correlation between platelet count and tumor size, while a negative correlation existed with survival and metastasis risk [69,90]. Histological analysis of HCC biopsies demonstrates that activated platelets cluster near tumor cells, utilizing GPIIb/IIIa, GPIb-IX-V, and P-selectin receptors to act as a “protective shield” against immune-regulated clearance [91,92].

Furthermore, platelets promote tumor growth, trans-endothelial migration, and epithelial–mesenchymal transition by releasing granule contents and directly interacting with tumor cells [65]. In both in vitro and in vivo studies, TGF-β derived from platelets has been shown to downregulate the tumor suppressor gene KLF6, consequently fostering tumor cell proliferation and cell cycle progression [93]. In vitro studies revealed that 5-HT enhances cell survival and proliferation by inhibiting autophagy in an m-TOR-independent manner [94].

Through their granule content of pro-angiogenic and growth factors, platelets foster a pro-tumoral niche and support tumor cell metastasis. Platelets facilitate the adherence of metastatic cells to the endothelium, trapping them with other immune cells, mostly via selectin-mediated interactions [95]. Moreover, the interaction between platelets and tumor cells is crucial in preventing detachment-induced apoptosis, which is a hallmark of metastasis [96]. Clinical studies show positive correlations between platelet counts and HCC recurrence or lack of response to chemotherapy, with levels dropping in responders after HCC treatment [97]. The bidirectional communication between cancer cells and platelets suggests that cancer cells influence platelet activation and morphology by secreting growth factors that bind to specific receptors on platelets surface. The close interaction between platelets and tumor cells, along with their physical interactions, raises the possibility of platelets serving as vectors for targeted drug delivery [69].

## 4. The Interplay between Platelets and Neutrophil Extracellular Traps in MASLD

As previously demonstrated in numerous preclinical animal models of chronic liver inflammation, platelets facilitate the homing of a variety of inflammatory effector cells to the site of inflammation, including neutrophils, macrophages, natural killer T (NKT) cells, and cytotoxic CD8^+^ T cells [98]. Moreover, another study on MASLD patients with increased inflammation markers and LPS plasma levels found increased inflammatory transcripts present in circulating platelets rather than in leukocytes, as expected. Miele et al. additionally showed that the increased intrahepatic platelet accumulation correlated with NAS score and intrahepatic NETs formation [99]. The increased accumulation of hepatic platelets and the development of liver NETs in the presence of low-grade endotoxemia raises the possibility that platelets might protect the liver by promoting local NETs formation against invading microorganisms.

Neutrophil infiltration within the liver is an important step mediating the transition from simple steatosis to statohepatitis. Neutrophils are one of the key players in innate immunity and work as part of the first line of defense against invading microorganisms. Although initially considered only an important component of the innate immune system, neutrophil extracellular traps were subsequently identified to be key players in various diseases characterized by sterile inflammation or to be involved in the activation of the hemostatic system [100]. NETs formation represents a distinctive form of neutrophil death and consists of a fibrous mesh of decondensed DNA intertwined with various nuclear and granular proteins that capture and neutralize microorganisms in order to prevent their spread [101]. NETs are released within the liver as a result of infection, ischemia, and sterile inflammation. NETs, while protective, may also have a cytotoxic effect on host cells [102]. 

**Figure 1:**
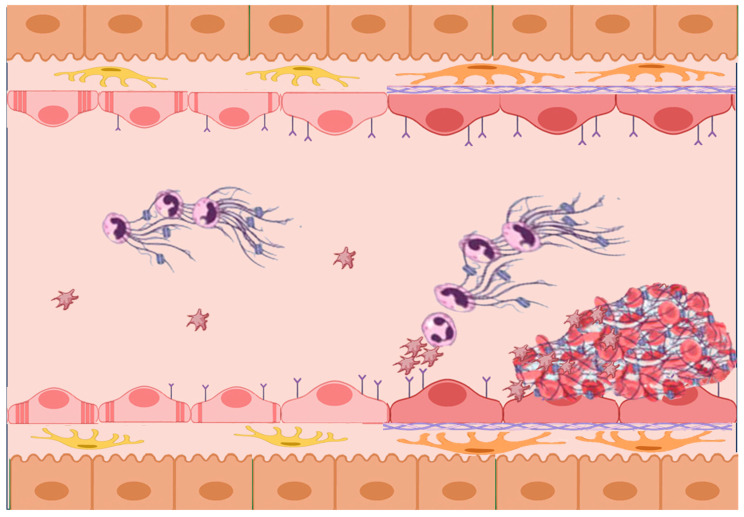
The interplay between endothelial cells, platelets, and NETs. In early MASLD stages, dietary factors induce LSEC capillarization, triggering pro-inflammatory responses in KCs and activating HSCs. Capillarized LSECs display a pro-inflammatory phenotype with adhesion molecule overexpression and increased cytokine production. Platelet interaction enhances leucocyte recruitment, NETs formation, and liver inflammatory foci. Platelets contribute to MASLD progression by fostering fibrosis, microthrombosis, parenchymal extinction, and fibrotic healing. They also promote tumor growth, epithelial-mesenchymal transition, and tumor cell metastasis.

Platelets facilitate NETs formation by recruiting and activating neutrophils through ligand-receptor interactions and chemokine secretion, primarily via the interaction between platelet P-selectin and neutrophil PSGL-1 (see Figure 1) [64,103,104]. The recruitment of neutrophils is further enhanced by the secretion of various cytokines, chemokines, and growth factors such as platelet factor 4 (PF4), IL-1, PDGF, platelet-activating factor, thromboxane A2 and 5-HT, by platelets [105].

On the other hand, histone and polyphosphate components from the NETs matrix contribute to both platelet and coagulation activation, promoting an anti-fibrinolytic microenvironment that accentuates liver damage [106,107]. Disrupting NETs formation has been shown to decrease collateral tissue damage and microcirculation thrombosis without impairing bacterial clearance [108]. The correlation between a procoagulant state and NETs formation was outlined in a study by Jingwen Du et al., where NETs isolated from MASH increased the procoagulant activity compared with controls [109].

Within the natural history of MASH, the role of NETs seems to be more prominent in the early stages of the disease, as proven by the study of Zang et al., which showed that neutrophil depletion led to decreased inflammation and reduced liver injury only in the early stages, effects that were lost as the disease progressed [110]. During the early stages, NETs formation relies on S1P receptor 2 signaling and has a pro-inflammatory effect that accentuates disease progression [111]. Moreover, NETs can modulate the inflammatory milieu in MASH by attracting monocyte-derived macrophages [112]. However, in later stages, NETs contribute to MASH-to-HCC transitions and HCC metastasis [5].

There are few hypotheses regarding the triggers of NETs formation during MASH progression. In a study by Wu J. et al., the initiating event for NETs formation in an early MASH mouse model was considered the changes in linoleic acid and γ-linolenic acid levels, suggesting that fatty acids are involved in regulating NETs development [113]. Another factor that might favor their formation during MASLD is the stretching of LSECs caused by the mechanical compression exerted by the fat-laden hepatocytes, which activates Notch-dependent neutrophil chemotaxis [17]. 

Apart from favoring sterile inflammation and a procoagulant state, NETs can also contribute to fibrosis development and progression by activating HSC through reactive oxygen species and MPO production [114,115]. In addition to this, the intraluminal development of this web-like structure (NETs) within the sinusoid increases the blood share stress at this level, which together with the microthrombosis, mechanical compression of the fat-laden hepatocytes and the peri-sinusoidal fibrosis, increase presinusoidal resistance and favor the development of portal hypertension in MASLD [116,117].

## 5. Coagulation Disturbances in MASLD

Although MASLD is linked to an increased risk of cardiovascular disease, a direct link between MASLD and global coagulation disturbances is yet to be proven. Among the potential contributing factors to the hypercoagulable state from MASLD, one can mention the ongoing inflammation, endothelial dysfunction, elevated circulating chromatin substances such as NETs, as well as hypertriglyceridemia, a prothrombotic structure of the fibrin clot together with the prothrombotic features induced by the associated comorbidities (i.e. insulin resistance, diabetes mellitus and obesity) [107,118,119,120].

The close interplay between inflammation and thrombosis has prompted the introduction of the term “immunothrombosis”. This concept is rooted in the observation that during inflammation, elevated levels of C-reactive protein promote interactions between monocytes and endothelial cells, increase PAI-1 activity, and induce tissue factor formation while also suppressing natural anticoagulant systems [121]. The active involvement of endothelial cells in the inflammatory process consists of the production of several molecules, including vWF, factor VIII, and fibrinogen, which are at the same time inflammatory and coagulation factors and which show increased levels in MASLD [122]. Inflammatory mediators like IL1 and TNF-α facilitate the release of vWF from activated endothelial cells [123]. The generated vWF is then partially secreted into circulation. The remaining part is attached to the endothelial surface where it can bind to other vWF molecules and platelets, favoring microthrombosis, or can be cleaved by ADAMTS13 [124]. Moreover, as inflammatory cytokines (i.e., IL6, TNF-α) whose levels are elevated in MASLD inhibit ADAMTS13 secretion and activity, an increased number of vWF multimers becomes available and, together with the rise in FVIII, enhances the prothrombotic status [125,126]. Endothelial dysfunction, through the decreased levels of NO production, can additionally contribute to the prothrombotic status from MASLD as NO regulates various blood vessels functions (vasodilatation, protection against vascular damage) and prevents platelet aggregation and leukocyte adhesion [127].

Another factor responsible for the hypercoagulable state from MASLD is the development of NETs, which has been shown to contribute to a reduced clotting time, a significant increase in thrombin-antithrombin complex (TAT) generation and fibrin formation, and whose effects can be blunted through DNase I pretreatment [109]. NETs play a role both in sustaining a hypercoagulable state in MASH and in accelerating the transition from MASH to liver fibrosis [109]. Elevated plasma triglyceride levels can contribute not only to MASLD development but also to the hypercoagulable state that characterizes it. Notably, hypertriglyceridemia has been associated with increased levels of clotting factors II, VII, IX, X, and XI [128]. Among these factors, factor VII holds particular significance as it is the initial enzyme in the extrinsic clotting cascade and whose levels increase in the presence of lipoprotein lipase in MASLD patients with high levels of circulating triglyceride-rich lipoproteins [129]. 

When compared to healthy controls, MASLD patients show changes in ex vivo clot kinetics, as proven by increased clot strength and delayed clot lysis in the presence of t-PA [130]. As the changes in thromboelastography-assessed clot kinetics were not correlated with histological severity of liver injury in MASLD patients, the elevated cardiovascular risk that characterizes these patients appears to be attributable to obesity-related metabolic dysfunction rather than hepatic disease [130]. Another possible mechanism explaining the changes in clot kinetics is represented by the fibrinogen molecule’s oxidative modifications found in obese patients [131].

The severity of homeostatic changes increases over the MASLD spectrum, but prothrombotic features seem to be driven mostly by concurrent comorbidities like insulin resistance, diabetes mellitus, and obesity. Insulin resistance is significantly associated with enhanced platelet reactivity and increased platelet adhesion following vascular injury, impairing insulin’s inhibitory effects on platelets due to abnormal adipokine content, namely resistin, leptin, PAI-1 and retinol-binding protein 4 [132].

Hyperglycemia stimulates platelet hyperreactivity, as indicated by increased aggregation, fibrinogen binding, and thromboxane A2 (TXA2) generation. In diabetic patients, the enzyme aldose reductase may cause platelet hyperreactivity by stimulating TxA2 production and release, resulting in increased platelet activation [133]. Furthermore, platelets from diabetic individuals are less sensitive to the antiaggregatory effects of insulin, NO, and PGI2 [134]. Since antiplatelet effects are associated with enhanced platelet NO synthesis, the reduced protective effects of aspirin against thrombotic events in type 2 diabetes mellitus may be explained, at least in part, by the reduced sensitivity to NO signaling [135]. High glucose concentrations are also associated with mitochondrial dysfunction and platelet damage, as well as mitochondrial membrane potential dissipation and caspase-3 activation, which lead to apoptosis in subsets of platelets [136]. Platelet hyper-reactivity in MASLD patients can also be determined by the activated coagulation cascade that favors thrombin production, which not only cleaves fibrinogen into fibrin but also represents the most potent platelet activator via proteinase-activated receptor (PAR) 1-4 signaling [75]. This observation is supported by a murine study showing that the use of thrombin inhibition with dabigatran in a mouse model with a high-fat diet can attenuate body weight gain and reduce hepatic fibrin deposition, inflammation, and hepatocyte injury, thus limiting MASLD progression [137]. Although in lean healthy people, insulin exerts an antithrombotic effect as it inhibits platelet-collagen and subsequent platelets-platelets interaction. However, in obese patients, this effect disappears [138]. 

Obesity has been linked to decreased fibrinolytic response to thrombosis, platelet hyperactivity, and resistance to anti-aggregating properties of insulin, aspirin, prostacyclin, and NO [139]. Platelets from obese patients are hyperactive, as seen by increased aggregation in response to different agonists such as collagen, adenosine diphosphate, arachidonic acid, and thromboxane A2 [140,141]. Leptin, an adipokine that correlates with MASH severity and promotes arterial thrombosis in a platelet leptin receptor-dependent manner, is a potential link between platelets, MASLD, and obesity [142,143]. Changes in additional prothrombotic cytokines, such as PAI-1 and hs-CRP, may represent a plausible mechanism linking obesity and clot kinetics [144]. Visceral adipocytes produce prothrombotic PAI-1 and are intimately linked to visceral adiposity in MASLD, as well as to the severity of steatosis and insulin resistance in humans and mice models of obesity [122,145]. 

## 6. Implications and Future Directions

As the gatekeeper of the hepatic microenvironment, LSECs exhibit multiple functions owing to their distinctive structure and anatomical location. Given the limited understanding of intercellular communication in the hepatic sinusoidal microenvironment, the recent advancements in single-cell technology offer an opportunity to delve deeper into the intercellular crosstalk among various LSECs, leading to a better comprehension of MASH and liver fibrosis.

Neutrophils play significant roles in various processes involved in liver disease pathogenesis, making them appealing targets for therapeutic strategies. Targeting NETs in MASLD has the potential to improve therapy and avoid progression to more severe forms such as MASH and cirrhosis. Moreover, addressing NETs could mitigate the substantial burden of cardiovascular disease morbidity and mortality linked to hypercoagulable and prothrombotic alterations. Further studies are essential for the development of new drugs targeting NETs development in MASLD. These drugs could be utilized alone or in conjunction with existing therapeutic approaches, including anti-inflammatory agents, NETs-degrading enzymes, anticoagulants, or antiplatelet medications, to enhance liver health and diminish the risks of liver cirrhosis and HCC.

Moreover, the involvement of platelets in mediating the interaction between neutrophils and endothelial cells, as well as in favoring liver fibrosis and thrombosis, suggests that antiplatelet treatment may present a potential benefit in delaying or even counteracting the progression of liver disease in MASH patients. Moreover, as the dual roles of platelets in different stages of liver disease are also reflected by the contrasting granule content (pro and anti-inflammatory, pro and anti-fibrotic, pro and anticoagulant), one could speculate a possible differential degranulation of platelets in accordance to different factors from the microenvironment. This supports further research that can lead to a better selection of patients who would benefit most from antiplatelet treatment.

In conclusion, among the residential cells, LSECs play a pivotal role in the initiation of inflammation, fibrosis, and angiogenesis within the liver, their capitalization and endothelial dysfunction together with their interaction with platelets and neutrophils being key events in the pathogenesis of MASH and MASH related-HCC. Further studies evaluating the effect of antiplatelets and anti-NETs medication in delaying or even counteracting the progression of liver disease in MASH patients are urgently needed.
